# Supplementary material for: Clinician Assessment Tools for Patients with Diabetic Foot Disease: A Systematic Review
Source: J Clin Med. 2020 May 15;9(5):1487. doi: 10.3390/jcm9051487 (PMC7291260; doi:10.3390/jcm9051487)
Supplement: Supplementary file 1 [file jcm-09-01487-s001.pdf]

**Table 1.** Characteristics of the Clinician assessment tools included in the study.

| SCALE | STUDY SUBJECTS | DIMENSIONS | SCORE | DESCRIPTION |
|---|---|---|---|---|
| 60-second Inlow's assessment tool | N=69. All with diabetes. Long-term care N=43<br>Mean age 88±8<br>21 male.<br>Dialysis group N=26<br>Mean age 69±10<br>10 male. | 1. Look tests (skin, nails, deformity, footwear)<br>2. Touch tests (temperature, range of motion)<br>3. Assess tests (sensation, pulses, rubor, erythema) | Each test is graded numerically. Patient-rated questions are included in the total score.<br>Maximum score= 23 | Test each foot independently<br>Dimensions were not well-defined. |
| American Diabetes Association System(ADA) | N=364. All with diabetes. Mean age=65±10.6<br>48.6% male | 1. Inspection<br>2. Neurologic assessment<br>3. Vascular assessment | Patients are allocated in risk groups from 0 to 4 according to examination and history | Test bilaterally. The authors did not define clear dimensions but risk groups |
| Basic Foot Screening Checklist (BFSC) | N=500 (112 were taken for reliability study). All with diabetes. | 1. Foot problems<br>2. Foot pulses<br>3. Neuropathy<br>4. Footwear<br>5. Education<br>6. Self care capacity | Test results are transcribed into whole values and addition is made for the total score.<br>Maximum score= 24 | Test bilaterally |
| Chronic Lower Extremity Ulcer Score | N=2019. N=1000 with diabetes.<br>Mean age=70<br>58% male | 1. Multiple ulcer<br>2. Wound area<br>3. Wound history<br>4. Non-palpable pulses | Test results are transcribed into whole values and addition is made for the total score.<br>Maximum score= 4 | Test each foot independently |
| Clinical Signs and Symptom Checklist (CSSC) | N=64. All with diabetes.<br>Mean age=55±11.4<br>77% male | Thirteen items regarding DFU infection | Test results are transcribed into whole values and addition is made for the total score.<br>Maximum score= 13 | Test each DFU independently |
| Curative Health Services system(CHS) | N=27,630. All with diabetes.<br>Mean age=64 to 65<br>51 to 55% male | 1. Wound age<br>2. Wound size<br>3. Wound age | Test results are transcribed into whole values and addition is made for the total score.<br>Maximum score= 3 | Test each DFU independently |
| Depth, extent of bacterial colonization, phase of healing and associated etiology system (DEPA) | N=137. All with diabetes. Mean age=61±17<br>58% male. | 1. Depth of the ulcer<br>2. Extent of bacterial colonization<br>3. Phase of ulcer<br>4. Associated etiology | Test results are transcribed into whole values and addition is made for the total score.<br>Maximum score= 12 | Test each DFU independently |
| Diabetic Foot Infection Wound Score (DFIWS) | N=371. All with diabetes.<br>Mean age=59<br>61% male. | 1. Wound purulent discharge<br>2. Wound measurements<br>3. Signs and symptoms of inflammation | Test results are transcribed into whole values and addition is made for the total | Test each foot independently |

| | | | | |
|---|---|---|---|---|
| | | | score.<br>Maximum score= 49 | |
| Diabetic foot risk assessment (DIAFORA) | N=293. All with diabetes.<br>Mean age=67.6±11.7<br>64.2% male | 1. Peripheral neuropathy<br>2. Foot deformity<br>3. PAD<br>4. Previous DFU or amputation<br>5. Multiple DFU<br>6. Infection<br>7. Gangrene<br>8. Bone involvement | Test results are transcribed into whole values and addition is made for the total score.<br>Maximum score= 41 | Test each foot independently |
| Diabetic Foot Ulcer Assessment Scale (DFUAS) | N=66. All with diabetes. Mean age=54<br>66,1% female. | 1. Depth<br>2. Size<br>3. Inflammation/Infection<br>4. Granulation tissue<br>5. Necrotic tissue<br>6. Maceration<br>7. Wound edge<br>8. Tunneling | Test results are transcribed into whole values and addition is made for the total score.<br>Maximum score= 48 | Test each DFU independently |
| Diabetic foot ulceration risk checklist (DFURC) | N=477. All with diabetes. Mean age=61.1±13.7.<br>52.2% male | 1. Course and complications<br>2. Neuropathy and vasculopathy<br>3. Ulcer<br>4. Deformity<br>5. Fungal infection | Test results are transcribed into whole values and addition is made for the total score.<br>Maximum score= 12 | Test each foot independently |
| Diabetic Neuropathy Examination (DNE) | N=60. All with diabetes.<br>24 male. | 1. Muscle strength<br>2. Reflexes<br>3. Sensation of index finger<br>4. Sensation of big toe | Test results are transcribed into whole values and addition is made for the total score.<br>Maximum score= 16 points | Test only right lower limb |
| Diabetic Ulcer Severity Score (DUSS) | N=137. All with diabetes. Mean age=61±17<br>58% male. | 1. Pulses<br>2. Probing-to-bone<br>3. Ulcer location<br>4. Presence of multiple ulcers | Test results are transcribed into whole values and addition is made for the total score.<br>Maximum score= 4 | Test each DFU independently |
| Early Neuropathy Scale (ENS) | N=113, 81 with pre-diabetes and neuropathy.<br>50 male.<br>Mean age=62.04 ± 1.33 | 1. Sensory loss testing<br>2. Ankle reflexes | Test results are transcribed into whole values and addition is made for the total score.<br>Maximum score=10 | Test bilaterally |
| Infectious Diseases Society of America (IDSA) -IWGDF classification | N=1,066. All with diabetes | Four clinical descriptions regarding DFU, infection, symptoms and systemic health | Only one score matching descriptions (uninfected, mild, moderate, severe) is given to the DFU. | Test each DFU independently |

| | | | | |
|---|---|---|---|---|
| Leg Ulcer Measurement Tool (LUMT) | N=22. Not all with diabetes.<br>Mean age=71±14<br>41% male | 1. Clinician-rated domains (14 items regarding DFU appearance, infection and edema)<br>2. Patient-rated domains (3 items regarding pain and quality of life) | Test results are transcribed into whole values and addition is made for the two domains independently. Total score is valid as well.<br>Maximum score= 68 | Test each foot independently plus patient-rated domains |
| Michigan Neuropathy Screening Instrument (MNSI) | N=125. All with diabetes. Mean age=58.7 ± 10.2<br>55 male. | 1. Questionnaire<br>2. Examination | Test results are transcribed into whole values and addition is made for the total score.<br>Maximum score= 15 and 8 | Test bilaterally. Dimensions were not well-defined |
| Modified International Working Group on the Diabetic Foot System(IWGDF) | N=364. All with diabetes. Mean age=65±10.6<br>48.6% male | 1. Neuropathy<br>2. Peripheral arterial disease<br>3. Deformity<br>4. Presence of ulcer<br>5. Infection | Patients are allocated in risk groups from 0 to 6 according to examination and history | Test bilaterally. The authors did not define clear dimensions but risk group. |
| Modified Toronto Clinical Neuropathy Scale (mTCNS) | N=113, 81 with pre-diabetes and neuropathy.<br>50 male.<br>Mean age=62.04 ± 1.33 | 1. Symptom scores<br>2. Sensory test scores | Test results are transcribed into whole values and addition is made for the total score.<br>Maximum score=33 | Test bilaterally |
| Neuropathy disability score (NDS) | N=60. All with diabetes.<br>24 male. | 1. Muscle strength<br>2. Tendon reflexes<br>3. Touch, vibration, joint position, pinprick | Test results are transcribed into whole values and addition is made for the total score.<br>Maximum score=10 | Test bilaterally |
| Neuropathy Impairment Score in the Lower Limbs (NIS-LL) | N=113, 81 with pre-diabetes and neuropathy.<br>50 male.<br>Mean age=62.04 ± 1.33 | 1. Power grading<br>2. Sensory testing<br>3. Reflex score | Test results are transcribed into whole values and addition is made for the total score.<br>Maximum score= 88 points | Test bilaterally |
| Non healing, exudates, red tissue, debris, smell criteria (NERD) | N=112 (67.6% with diabetes)<br>Mean age=66<br>60.4% male | 1. Non-healing<br>2. Exudate<br>3. Red friable tissue<br>4. Debris<br>5. Smell | Test results are transcribed into whole values and addition is made for the total score.<br>Maximum score= 5 | Test each DFU independently |
| Perfusion, extent, depth, infection and sensation scale (PEDIS) | N=364. All with diabetes. Mean age=66±12<br>219 male. | 1. Perfusion<br>2. Extent<br>3. Depth<br>4. Infection<br>5. Sensation | Test results are transcribed into whole values and addition is made for the total score.<br>Maximum score= 12 | Test each DFU independently |

| | | | | |
|---|---|---|---|---|
| Photographic Wound Assessment Tool (PWAT) | N=68. N=18 with diabetes. Mean age= 59.8 | 1. Size<br>2. Depth<br>3. Necrotic tissue type<br>4. Necrotic tissue amount<br>5. Granulation tissue type<br>6. Granulation tissue amount<br>7. Edges<br>8. Periulcer skin | Test results are transcribed into whole values and addition is made for the total score.<br>Maximum score= 32 | Test each DFU independently |
| Pressure Ulcer Scale for Healing (PUSH) | N=29. All with diabetes.<br>Mean age=54±11<br>66.6% male | 1. Length x Width<br>2. Amount of exudate<br>3. Tissue type | Test results are transcribed into whole values and addition is made for the total score.<br>Maximum score=17 | Test each DFU independently |
| Queensland High Risk Foot Form (QHRFF) | N=19 to 43. All with diabetes.<br>Mean age=68 to 70±15<br>78 to 90% male | High risk form consisting in >20 questions and tests | This is not a numerical scale. Patients are allocated in risk groups according to descriptions | Test bilaterally |
| Scottish Foot Ulcer Risk Score (SFURS) | N=3526. All with diabetes. Mean age=64.7 | 1. High risk<br>2. Moderate risk<br>3. Low risk | This is not a numerical scale. Patients are allocated in risk groups according to descriptions | Test bilaterally. There are not well-defined dimensions but risk groups |
| Scottish Intercollegiate Grouping Network System (SIGN) | N=364. All with diabetes. Mean age=65±10.6<br>48.6% male | 1. Protective sensation<br>2. Deformity<br>3. History of ulceration o/and amputation | Patients are allocated in risk groups from 0 to 4 according to examination and history. | Test bilaterally. The authors did not define clear dimensions but risk groups |
| Seattle Risk Score | N=364. All with diabetes. Mean age=65±10.6<br>48.6% male | 1. A1C<br>2. Vision quality<br>3. History of DFU or amputation<br>4. Monofilament insensitivity<br>5. Fungal infections | Patients are allocated in risk groups from 0 to 4 according to examination and history. | Test bilaterally |
| Sepsis, arteriopathy, denervation System (SAD) | N=105. All with diabetes.<br>Mean age=57.6<br>60.6% male | 1. Area<br>2. Depth<br>3. Sepsis<br>4. Arteriopathy<br>5. Denervation | Test results are transcribed into whole values and addition is made for the total score.<br>Maximum score= 15 | Test each foot independently |
| Site, Ischemia, Neuropathy, Bacterial Infection, and Depth score(SINBAD) | N=137. All with diabetes. Mean age=61±17<br>58% male | 1. Site<br>2. Ischemia<br>3. Neuropathy<br>4. Bacterial infection<br>5. Area<br>6. Depth | Test results are transcribed into whole values and addition is made for the total score.<br>Maximum score= 6 | Test each DFU independently |
| Size, temperature, osteomyelitis edema, exudate, smell criteria (STONEES) | N=112 (67.6% with diabetes)<br>Mean age=66<br>60.4% male | 1. Size<br>2. Temperature<br>3. Bone | Test results are transcribed into whole values and addition is made for the total | Test each DFU independently |

| | | | | |
|---|---|---|---|---|
| | | 4. New breakdown<br>5. Edema/erythema<br>6. Exudate<br>7. Smell | score.<br>Maximum score= 7 | |
| Toronto Clinical Neuropathy Scale (TCNS) | N=89. All with diabetes. Mean age=54.2 ± 10.2,<br>65 male. | 1. Symptom scores<br>2. Reflex scores<br>3. Sensory test scores | Test results are transcribed into whole values and addition is made for the total score.<br>Maximum score=16 | Test bilaterally |
| Total Neuropathy Score (Clinical) | N=113, 81 with pre-diabetes and neuropathy.<br>50 male.<br>Mean age=62.04 ± 1.33 | 1. Motor and autonomic symptoms<br>2. Pin sensation<br>3. Vibration sensibility<br>4. Strength<br>4. Tendon reflexes | Test results are transcribed into whole values and addition is made for the total score.<br>Maximum score=24 | Test bilaterally |
| United Kingdom Screening Test (UKST) | N=125. All with diabetes. Mean age=58.7 ± 10.2<br>55 male. | 1. Symptom score<br>2. Sign score | Test results are transcribed into whole values and addition is made for the total score.<br>Maximum score= 19 points | Test bilaterally<br>Each test is graded differently (e.g: "present" might be =0 or =1) |
| University of Texas classification | N=360. All with diabetes. Mean age=53.9±10.4<br>68,6% male. | 1. Depth<br>2. Infection | One single score is obtained from a 4x4 matrix. Grades are from 0 to 3 + from A to D. (e.g: 2C) | Test each DFU independently |
| University of Texas System(UT) | N=364. All with diabetes. Mean age=65±10.6<br>48.6% male | 1. Protective sensation<br>2. Deformity<br>3. History of ulceration o/and amputation | Patients are allocated in risk groups from 0 to 4 according to examination and history. | Test bilaterally. The authors did not define clear dimensions but risk groups. |
| Utah Early Neuropathy Scale (UENS) | N=215. All with diabetes and pre-diabetes.<br>129 with neuropathy.<br>Mean age 57.8±7.1<br>54.2% female.<br>86 without neuropathy. Mean age 55.8±9.7<br>50% female. | 1. Motor examination<br>2. Pin sensation<br>3. Allodynia/hyperesthesia<br>4. Large fiber sensation<br>5. Deep tendon reflexes | Test results are transcribed into whole values and addition is made for the total score.<br>Maximum score= 42 | Test bilaterally |
| Wagner's classification | N=137. All with diabetes. Mean age=61±17<br>58% male. | 0. Intact skin<br>1. Superficial ulcer<br>2. Ulcer reaching tendon, joint or bone<br>3. Grade 2 plus infection<br>4. Gangrene of portion or all forefoot<br>5. Gangrene or dysvascularity of the entire foot | Only one score from 0 to 5 is given to the DFU. | Test each DFU independently |
